# Cricothyroidotomy in extreme emergency: A case report of a real-life experience in three steps and less than 30 s using a single blade

**DOI:** 10.1016/j.ijscr.2024.109526

**Published:** 2024-03-15

**Authors:** S. Morand, A. Gleizal

**Affiliations:** Maxillofacial Surgery Department, Lyon Croix Rousse Hospital, 103 Grande rue de la Croix Rousse, 69004 Lyon, France

**Keywords:** Case report, Cricothyroidotomy, Emergency

## Abstract

**Introduction and importance:**

Cricothyroidotomy is often the last resort when conventional ventilation devices prove ineffective. The conventional procedure that involves several steps and requires the completion of a preoperative checklist. This report describes a novel approach to cricothyroidotomy, allowing quick access to the cricothyroid membrane in fewer steps.

**Case presentation:**

We present the case of a 26-year-old male with Schimmelpenning syndrome, exhibiting significant anatomical deformity. Following surgery for temporomandibular joint replacement, the patient developed a hematoma requiring urgent intervention. During nasotracheal intubation, the patient experienced a significant drop in oxygen saturation, which required prompt cricothyroidotomy. The procedure was performed in less than 30 s using a single blade for incising the tissues and the surgeon's hands for dissection and retraction. The procedure resulted in immediate recovery of the patient's oxygen saturation.

**Clinical discussion:**

In contrast to previously reported multi-step procedures, this study reports a simpler three-step cricothyroidotomy. The technique involves a vertical skin incision, blunt dissection using the surgeon's fingers, and a horizontal incision on the cricothyroid membrane. The procedure was executed with the patient in a semi-reclined position, optimizing time efficiency.

**Conclusion:**

This case highlights the efficacy of a rapid cricothyroidotomy technique in extreme emergencies. The presented technique requires minimal instrumentation and can be completed quickly in an emergency situation, even in the presence of anatomical variations.

## Highlights

• We present a quick cricothyroidotomy technique executed in less than 30 s.
• The technique required the use of a single blade and the surgeon's hands.
• The technique proved effective even in a case of atypical anatomy.
• The procedure could be performed in a semi-reclined position, improving efficiency.

## 1 Introduction

**Fig. 1:**
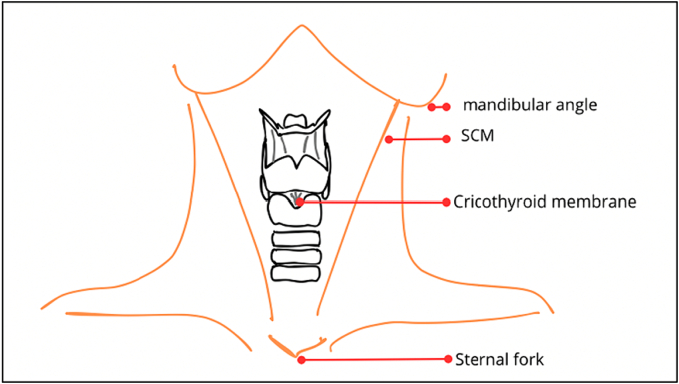
Anatomy of the larynx.

Securing the airway is a crucial step in the primary care of any patient. An analysis of 184 studies by the NAP4 project indicated that airway complications occur in one per 22,000 general anaesthetics [1]. There is a lack of published literature regarding the optimal timing for cricothyroidotomy. A consensus statement by the Difficult Airway Society, Eastern Association for the Surgery of Trauma, and American College of Emergency Physicians suggests that cricothyroidotomy should be performed after three failed attempts of endotracheal intubation. However, adverse events, including hemodynamic states and hypoxia can occur even after a single failed intubation attempt and may negatively impact patient outcomes [2]. In this context, the maxillofacial surgeon must be proficient in performing an emergency cricothyroidotomy, which involves opening the cricothyroid membrane (Fig. 1). This procedure is often the last resort when conventional ventilation devices prove ineffective. A review of emergency airways over a 6-year period reported that 24 of the 34 patients underwent emergency tracheotomy, while 10 underwent cricothyrotomy [3]. Several authors have investigated the execution of this maneuver [4], with differing opinions on the choice between surgical and percutaneous techniques [5]. The percutaneous approach requires specific equipment and the knowledge of its application. The surgical technique involves considerations, such as the orientation (vertical or horizontal) of the skin incision [6] and its positioning [7]. In cases of acute respiratory distress, the surgical teams are often unprepared and lack the necessary equipment. Consequently, the surgeon must rely on the familiar classic tracheotomy technique, which is time-consuming and leads to critical delays.

Herein, we present a real-life experience involving a 26-year-old patient with a rare malformative syndrome who developed acute respiratory distress and subsequently underwent a cricothyroidotomy in an extreme emergency at the maxillofacial surgery department of Croix Rousse hospital, Lyon, France. Despite the atypical anatomy, the procedure was remarkably performed in less than 30 s using a single instrument: a blade.

## 2 Case report

The work has been reported in line with the SCARE criteria [8].

We present a case of a 26-year-old male patient diagnosed with Schimmelpenning syndrome, a rare malformative syndrome characterized by a significant deformity of the left hemiface and thorax (Fig. 3). The patient exhibited a complete lack of mouth opening (0 mm).

**Fig. 2:**
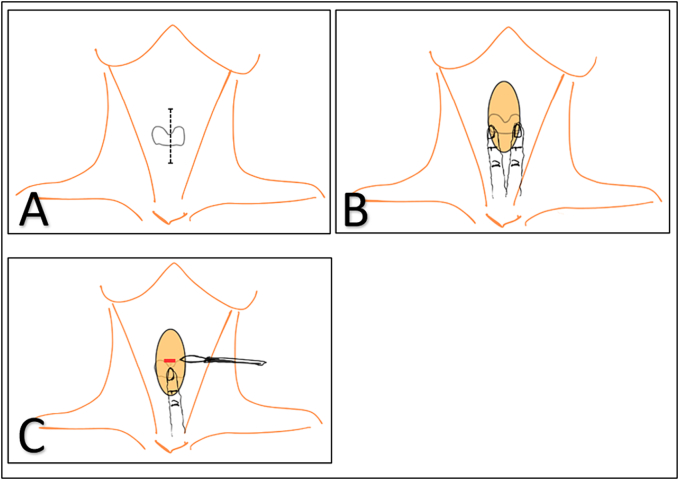
Fast cricothyroidotomy in three short steps. A: First step involves making an incision in the correct position. B: Second step involves finger dissection. C: Third step involves the membrane incision.

The patient was initially managed in the Maxillofacial Surgery Department, where a specialized left custom-made temporomandibular joint prosthesis was placed. General anesthesia was administered with fibro-vigil naso-tracheal intubation without any complications. Risdon and preauricular incisions were used for prosthesis placement, with two drains inserted after the surgery. The immediate postoperative period was uneventful, and due to the patient's syndrome, postoperative monitoring was performed in a continuous care unit. The drains were removed on the third postoperative day. The skin was edematous but not fluctuant on palpation. The patient was shifted from the continuous care unit on the fourth postoperative day. However, on the fifth postoperative day, the patient reported pain at the surgical site. A control computed tomography (CT) scan was performed, revealing a cervical collection measuring 5 cm in width and 6.5 cm in height, with a right airway laterodeviation (Fig. 4). Initially, the sutures at the surgical site were removed to facilitate drainage, but this had no effect on the collection. Subsequently, the patient was fasted, and a drainage procedure was performed in the operating room under general anesthesia, necessitating a new fibro-vigil nasotracheal intubation, initiated at 09:48 AM. At the time of initiation of the procedure, the patient showed no signs of airway distress, resulting in a decision to proceed with nasotracheal intubation instead of a tracheostomy. In a semi-reclined position, light sedation was achieved through regular injections of remifentanil. During this intubation attempt, difficulties arose in exposing the glottic region because of progressive edema affecting the entire glottal system and rightward laryngeal deviation. The vocal cords were nearly invisible, making intubation impossible. After 32 min, the patient experienced a sudden drop in oxygen saturation from 100 % to 35 % between 10:22 AM and 10:24 AM (Fig. 5). Immediately, the cervical scar was opened with a blade, resulting in the evacuation of a large super-infected hematoma. Subsequently, cricothyroidotomy was performed in three steps (Fig. 2). Initially, the cricoid was identified and a vertical 10-cm skin incision was made, centered on the cricoid but without taking any other anatomical landmarks into consideration, using a No. 15 blade. The soft tissues were separated around the cricoid using the index and middle fingers of the left hand, while the right hand continued to incise the tissues in a vertical direction until resistance from cricoid was felt under the blade. At this point, the left index finger was used to identify the cricoid cartilage and was positioned on the cricothyroid membrane. This provided guidance to the right hand, holding the blade, to make a horizontal incision on the cricothyroid membrane. A size 7 tracheostomy tube was placed directly into the trachea for ventilation. The entire procedure was performed by the Maxillo-facial surgeon of the Maxillofacial surgery department.

**Fig. 3:**
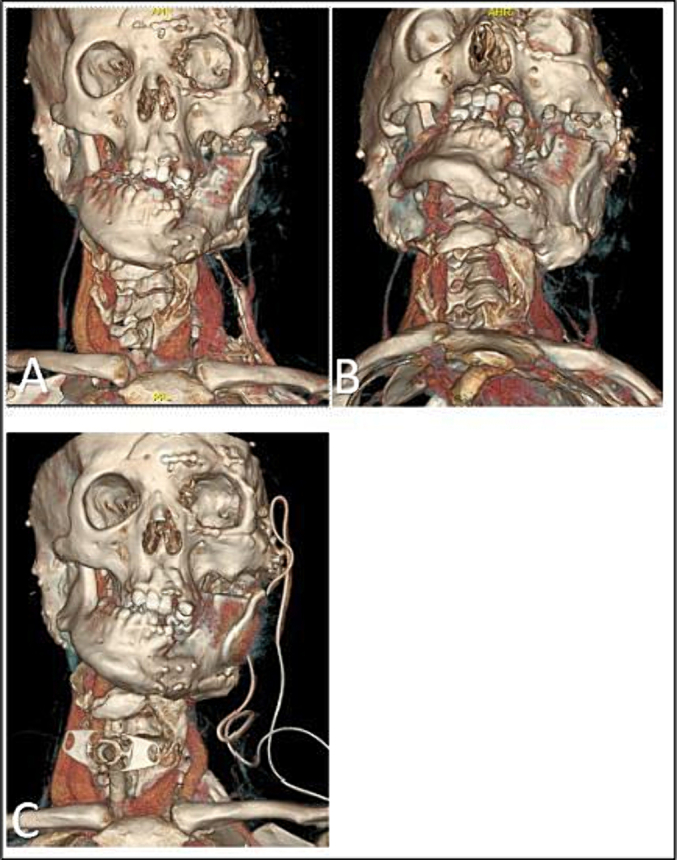
Patient anatomy. A: Frontal view. B: Inferior view. C: Frontal view showing the cricothyroidotomy position.

Due to the patient's unique anatomy and airway deviation, resulting from the postoperative hematoma (Fig. 3), cricothyroidotomy was performed on the right cervical side (Fig. 5). The patient rapidly regained satisfactory oxygen saturation. Following confirmation of ventilation, the tube was secured on each side with a single a suture. Subsequently, the planned surgical intervention was performed without any further episodes of reduced oxygen flow.

**Fig. 4:**
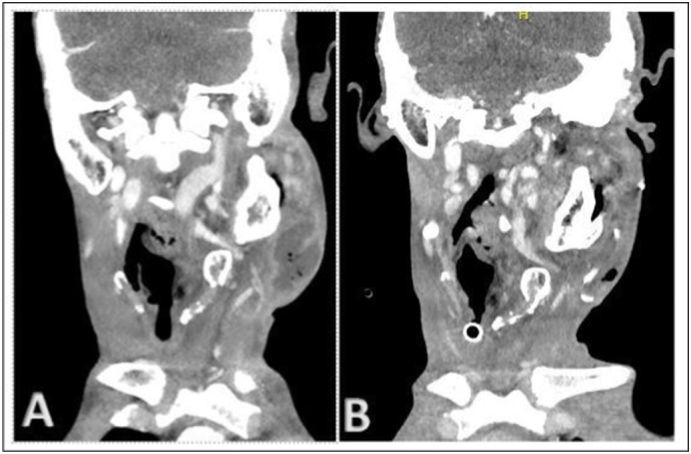
Cervico-facial scan before and after drainage. A: Left cervical collection before drainage. B: Collection drained along with cricothyroidotomy.

**Fig. 5:**
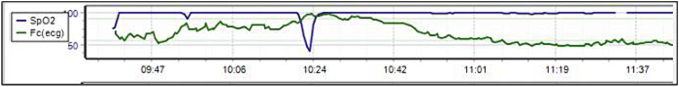
Evolution of oxygen saturation during intubation. At 10:22 AM, a significant drop in the oxygen saturation is noted. Cricothyroidotomy was performed at 35 % SpO_2_, resulting in the immediate recovery of oxygen saturation to 100 %.

Following the second surgery, the tube was left in place for 48 h. The patient spent 5 days in the continuous care unit. A fresh CT scan confirmed the proper positioning of the tracheostomy cannula and effective drainage of the left-sided collection. The cuff of the tube was deflated on the third day, and the tube was removed on the fifth day without any complications. During subsequent postoperative consultations, the patient presented no chronic complications, along with the absence of laryngeal stenosis.

## 3 Discussion

In an emergency, cricothyroidotomy is often considered the surgical airway of choice, preferred over an emergency tracheostomy. The procedure allows safe and effective airway management, with few contraindications, including tracheal transection, laryngeal crush injury, and obstruction distal to the cricothyroid membrane [9]. All the reported techniques involve neck extension, identification of the cricothyroid membrane, incision through the skin and cricothyroid membrane, and insertion of a cuffed tracheal tube under complete neuromuscular blockade and supplemental oxygen administration [10].

Several authors have detailed the cricothyroidotomy procedure, involving numerous steps and the completion of a comprehensive checklist before intervention [10]. For instance, guidelines from the Difficult Airway Society, Eastern Association for the Surgery of Trauma, and American College of Emergency Physicians suggest that cricothyroidotomy should be performed after three failed attempts of endotracheal intubation [2]. Practically, however, none of these elements come into play during emergency situations. The critical factor is reaching the correct anatomical location as quickly as possible. In literature, there is only one previous report of a similar three-step cricothyroidotomy procedure. However, the procedure was tailored specifically for a military setting and requires the use of a bougie [11]. Therefore, we present a method for performing cricothyroidotomy in under 30 s, utilizing only a single instrument and the surgeon's two hands in three simple steps.

Our recommended procedure involves a vertical skin incision centered on the cricoid cartilage. This allows incision in the direction of the principal vessels encountered, protecting them from inadvertent cuts of the blade. Additionally, by inserting the left index and middle fingers into the incision, the operator can perform blunt dissection up to the cricothyroid membrane without the need for a Farabeuf-type retractor. The membrane incision is placed horizontally, positioning the blade just above the upper edge of the cricoid cartilage and running along it to perforate the membrane. Furthermore, the procedure was performed with the patient in a semi-reclined position, eliminating the need to lay the patient down and saving valuable time. While several authors have advocated a four-step procedure using a tracheal hook to stabilize the larynx before inserting the tracheal cannula [12,13], we propose a three-step procedure using only a blade. This technique proved effective in securing the airway despite the patient's atypical anatomy.

The most common reported complication of cricothyrotomy is subglottic stenosis, but the evidence for this complication is nearly a century old. A review of the complications associated with cricothyroidotomy reported that the most common complications were injury to the cartilaginous structures and failure to obtain an airway, both resulting from an improper technique. Other complications included hemorrhage, pneumothorax, subcutaneous emphysema, airway stenosis, dysphonia, aspiration pneumonia, peristomal infection, and dysphagia [14].

## Consent

Written informed consent was obtained from the patient for publication of this case report and accompanying images. A copy of the written consent is available for review by the Editor-in-Chief of this journal on request.

## Ethical approval

In accordance with French law, review by an ethics committee was not required.

## Funding

The authors declare that they have no known competing financial interests or personal relationships that could have appeared to influence the work reported in this paper.

## Author contribution

First author: Writing, Operator, Data collection

Second author: Contribution, Validation

## Guarantor

First author.

## Conflict of interest statement

The authors declare that they have no known competing financial interests or personal relationships that could have appeared to influence the work reported in this paper.
